# Influencing Factors of Porosity and Strength of Plant-Growing Concrete

**DOI:** 10.3390/ma17010031

**Published:** 2023-12-20

**Authors:** Jiashi Cai, Chunying Shen, Ming Ye, Siyang Huang, Jinxing He, Ding Cui

**Affiliations:** 1 Faculty of Electric Power Engineering, Kunming University of Science and Technology, Kunming 650500, China; caijiashi0831@163.com (J.C.); cuiding0520@outlook.com (D.C.); 2 Sinohydro Bureau 6 Co., Ltd., Shenyang 110169, China; yeming@powerchina.cn (M.Y.); hejx@powerchina.cn (J.H.); 3 Faculty of Civil Engineering and Mechanics, Kunming University of Science and Technology, Kunming 650500, China

**Keywords:** plant-growing concrete, preparation process, porosity, compressive strength, fitting, stress–strain curve, planting test

## Abstract

A standardized preparation process is proposed in this study for achieving optimal strength and vegetative properties in vegetated concrete, using Yunnan red soil as a growth substrate for plants. The porosity of vegetated concrete is a crucial factor influencing plant growth, while compressive strength is a significant mechanical property. To assess the strength and porosity of vegetated concrete, different design porosities (22%, 24%, 26%, 28%) and cement-to-aggregate ratios (4, 5, 6, 7) were utilized in the preparation of vegetated concrete samples. The shell-making and static-pressure-molding methods were optimized for specimen preparation. Analyzing the stress–strain full curve characteristics of vegetation-type concrete under different influencing factors, an in-depth investigation into its failure mechanism was conducted. It was determined that the design porosity and cement content significantly impact the concrete’s performance, particularly in terms of 30-day compressive strength and effective porosity. Furthermore, an increase in the fly ash ratio led to an increase in porosity and a decrease in compressive strength, providing a certain guidance for optimizing concrete performance. Comparative analysis through vegetation experiments revealed that black rye grass exhibited favorable growth adaptability compared to other grass species.

## 1. Introduction

In recent years, due to production and sanitary sewage emissions, the ecological environment of coastal wetlands has been gradually damaged to varying degrees. A number of ecological problems have increased and estuarine revetment has become an important issue in the field of environmental protection and ecological restoration [1,2,3].

With the acceleration of global urbanization and the increasing prominence of environmental issues, the search for sustainable building materials and engineering solutions has become particularly important [4]. In contrast, vegetated concrete is an environmentally friendly slope protection material with strength and vegetation coverage. It meets the safety and protection requirements of engineering projects and exhibits excellent permeability and water retention properties that support vegetation growth. In practical applications, vegetated concrete serves as a protective measure, demonstrating environmental friendliness and biological compatibility. Therefore, many scholars have conducted in-depth research on its performance, mix ratio design, construction technology, and so on [5,6,7,8].

Currently, vegetated concrete can be categorized into two types based on the preparation process: porous and spray-based vegetation concrete. Porous vegetation concrete consists of two layers, with the lower layer composed of porous concrete made from coarse aggregate, cementitious materials, and a water-retaining agent. The upper layer includes soil, organic matter, and seeds [9]. Studies indicate that the compressive strength of porous vegetation concrete exceeds 10 MPa, with a continuous porosity ranging from 21% to 30%. In contrast, spray-based vegetation concrete’s 28-day unconfined compressive strength falls between 0.6 MPa and 1.11 MPa, with a continuous porosity of 33% to 43% [10]. Porous vegetation concrete, including coarse aggregate for support, meets the industry standard for vegetated concrete (JC/T 2557-2020 [11]) with a compressive strength exceeding 10 MPa and a continuous porosity between 21% and 30% [12].

In this experiment, the 30-day unconfined compressive strength values under different mix ratios range from 3.43 to 7.01 MPa. In comparison to ordinary concrete, the strength is approximately 15% to 25% due to a reduction in cement dosage by 1/3 to 1/4. The pore structure of porous materials can be characterized by several parameters, including pore size, connectivity, pore surface roughness, and porosity. Pore structure properties are direct factors affecting the basic properties of porous concrete, such as compressive strength and permeability [13]. The type, shape, and size of the coarse aggregates and the water–cement ratio are influencing factors that greatly affect the physical and mechanical properties of the planting concrete, such as porosity and strength [14]. Lian [15] and Jang [16] established a significant correlation between compressive strength and porosity in vegetated concrete. They proposed regression equations based on theoretical derivations to mathematically model the compressive strength of concrete under specific porosity conditions. Previous experiments [17,18] have primarily focused on the mechanical properties of the skeleton of vegetated concrete; however, in practical applications, vegetation is vital in maintaining slope stability, preventing and mitigating slope runoff, and achieving ecological sustainability. The effect of different plant species and growth stages on slope protection of plant-type concrete is very different [19].

Therefore, vegetative concrete, regarded as an eco-friendly material, incorporates coarse aggregates as a crucial constituent [20]. In contrast to other experiments, this study considers aggregates and vegetation as an integral entity for investigation. The unconfined compressive strength is used as an indicator of mechanical performance, while porosity is considered a primary parameter for microstructural analysis. By investigating the preparation process of vegetated concrete, including the mix design, construction, and maintenance processes, and conducting vegetation experiments using red soil, this study aims to explore the impact of different design porosities and cement-to-aggregate ratios on the performance of vegetated concrete. Additionally, a mathematical relationship equation between porosity and compressive strength is established. Through observing the plant growth conditions, suitable grass species for the Kunming region are selected. The aim is to provide a reference for plant concrete to meet the requirements of engineering safety protection in various applications and for river slope treatment [8].

## 2. Experimental Study

### 2.1. Raw Materials and Experimental Design

This experiment, considering the cost implications in practical engineering applications, aims to minimize the use of additional admixtures. Only single-sized coarse aggregates, cement, red soil, water-absorbing resin, and water were chosen for the experiment. Moreover, these materials are readily available, enhancing engineering efficiency.

This test employs Red Lion Brand M32.5-grade masonry cement produced by Yiliang Red Lion Cement Co., Ltd. (Kunming, China), The main chemical composition of cement is shown in Table 1. In comparison with other grades of cement, M32.5 cement is characterized by high strength, good workability, and excellent water retention properties. The relevant performance test results are presented in the table below, meeting the requirements of the national standard GB/T 3183-2017 [21].

The primary constituent of vegetated concrete is aggregates, typically with particle sizes ranging from 10 to 20 mm. A single particle size aggregate gradation is commonly employed to ensure sufficient porosity for vegetation growth, aiming for a porosity exceeding 15% [22,23]. The mechanical properties of vegetated concrete, including strength, porosity, permeability, and durability, heavily rely on coarse aggregates’ size, shape, and distribution [24,25]. In this experiment, the coarse aggregates consisted of stones with a continuous gradation ranging from 10 to 15 mm, without adding fine aggregates. The soil used in this study was collected from the vicinity of Songhua Dam Reservoir in Kunming City. After drying and crushing, the soil samples were sieved using a 5 mm mesh to obtain dry powder soil. Yunnan red soil, characterized by its significant iron and aluminum content and low organic matter, exhibits a sticky and heavy texture and is classified as slightly acidic soil. A superabsorbent polymer (SAP) with high water absorption capacity and excellent water retention properties was employed as the water-retaining agent for water retention purposes.

The soil porosity required for plant growth typically falls within the range of 40% to 60%. There has always been a mutually restrictive relationship between porosity and strength. While a smaller design porosity can ensure the strength of vegetation-type concrete, it may adversely affect the growth and germination of plant roots and also reduce the effectiveness of water filtration and air permeability. On the contrary, larger porosity is more inclined to be applied in highway landscape vegetation and eco-roof construction, where there are no strength grade requirements. Based on preliminary test results and pre-experimental studies, we determined that the suitable porosity range for vegetated concrete fell between 20% and 30%. At the same time, the cement-to-aggregate ratios ranged from 3.5:1 to 8:1. For this experiment, we selected design porosities of 22%, 24%, 26%, and 28%, corresponding to aggregate cement ratios of 4:1, 5:1, 6:1, and 7:1, respectively. Experimental groups were established, along with blank control groups, for vegetation experiments. A total of 48 specimens were prepared. The specific quantities of materials used can be found in Table 2.

### 2.2. Specimen Preparation

#### 2.2.1. Washing

Vegetated concrete typically utilizes crushed stones as coarse aggregates. Throughout the production, transportation, and storage processes, these crushed stones may inevitably carry impurities such as soil and stone dust. These impurities adhering to the surface of the crushed stones could significantly impact the performance of vegetated concrete. Therefore, before experimentation, cleaning the coarse aggregates by removing these impurities is essential.

Before cleaning the aggregates, use a sieve with a particle size of 10 to 15 mm for screening to remove obvious needle-shaped aggregates, obtaining a single-sized coarse aggregate. This step helps to prevent premature failure during mechanical performance testing of formed vegetation-type concrete specimens due to stress concentration between aggregates. After cleaning, lay the aggregates flat on the test site for air-drying. Due to the large quantity of crushed stones, it is impractical to dry them to a constant weight in the laboratory.

#### 2.2.2. Mixing

**Figure 1:**
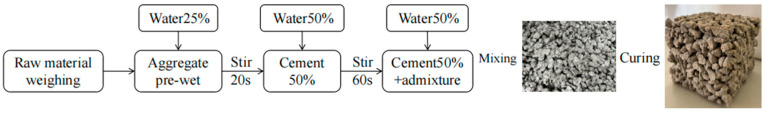
Preparation flow chart.

The mixing processes commonly employed in vegetated concrete include the one-time-feeding shell-making, and slurry-coating methods. In this study, the mixing of aggregates and cementitious slurry resulted in uneven distribution due to the use of a single-particle-size graded aggregate and the absence of fine aggregates for lubrication. The one-time feeding method led to agglomeration in the cementitious slurry. The initial mixing involved cement, water, and a water-retaining agent in the cement slurry-coating method. However, using a vertical forced mixer with a relatively slow mixing speed limited the water-reducing effect of the water-retaining agent. It hindered the adequate release of free water, resulting in severe agglomeration of the cement slurry. A more suitable mixing process was explored and optimized by reviewing the literature, summarizing experiences, and improving the shell-making method, as described in Figure 1.

#### 2.2.3. Preparation Process

Vegetated concrete employs three standard molding methods: manual tamping molding, vibration molding, and static pressure molding. These molding processes significantly influence the strength and porosity of vegetated concrete, requiring stricter control than ordinary concrete. The combination of manual tamping and layered pouring was found to be operationally feasible through comparative analysis and considering the high fluidity of the cementitious slurry. This method allows better control over the uniformity of the cementitious slurry enveloping the aggregates, minimizing settling at the bottom. The prepared vegetated concrete is divided into three layers and manually tamped into a 150 mm × 150 mm × 150 mm cubic mold.

After the preparation is completed, unconfined compressive strength tests are conducted on vegetation-type concrete specimens following a 30-day curing period. Common curing methods for vegetation-type concrete include standard concrete curing and natural curing. In practical engineering applications, vegetation-type concrete is typically cast on-site, often lacking the conditions for curing in specialized containers. To more authentically simulate the application of vegetation-type concrete in real-world scenarios, this experiment adopts the natural curing method. Shade nets are laid out at the testing site to prevent the surface stones from peeling off due to insufficient moisture during the curing and hardening process.

**Figure 2:**
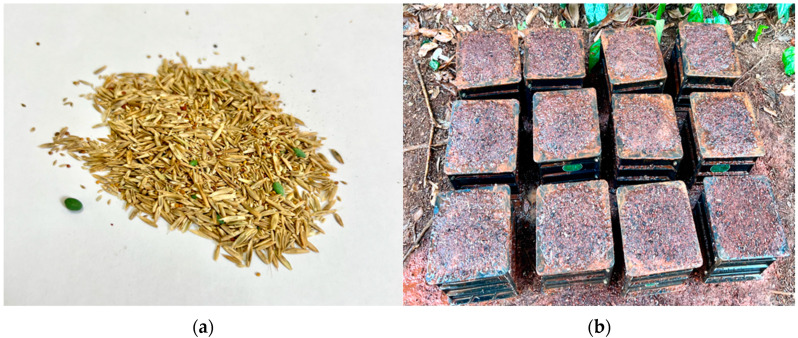
Plant growth test: (**a**) mixed grass seed; (**b**) test blocks to be planted.

After 7 days of natural conservation, a vegetation experiment was conducted utilizing the infiltration method, whereby nutrient substrate was introduced into the vegetation-type concrete framework. The pore-filling substrate comprised red soil, ferrous sulfate, and water. The surface nutrient substrate consisted of topsoil (50% soil + 50% humus soil) mixed with water in a 1:2 mass ratio. The selected plant species for mixed sowing, as depicted in Figure 2a, included black ryegrass, Cynodon dactylon, Festuca arundinacea, Dichondra repens, and Poa pratensis. The specific planting procedure involved: (1) injecting the prepared pore-filling substrate into the concrete framework using a cement gun; (2) uniformly sowing a combined grass seed mixture at a rate of 30–40 g/m^2^ after the substrate had solidified [26]; (3) covering with a 1 cm layer of surface nutrient soil. The completed experimental blocks after the vegetation test are illustrated in Figure 2b.

### 2.3. Testing Methods

#### 2.3.1. Stress–Strain Curve Test

The stress–strain curve of vegetated concrete provides a comprehensive representation of its mechanical behavior. It is a valuable tool for assessing fundamental properties, including compressive strength indicated by the stress at the curve’s peak, peak strain represented by the corresponding strain, elastic modulus reflected by the slope, and residual strength observed in the descending segment. Therefore, a detailed analysis of the uniaxial compressive stress–strain curve is essential for understanding the mechanical characteristics of vegetated concrete.

**Figure 3:**
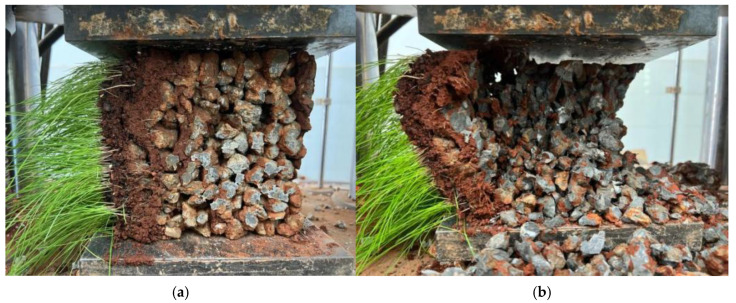
Failure process of planting concrete in compressive test: (**a**) specimen in unbroken state; (**b**) failure condition of specimen.

By the “Testing Code for Hydraulic Concrete” (SL/T352-2020) [27], the uniaxial compression specimens of vegetated concrete were prepared in the form of 150 × 150 × 150 mm cubes. The following steps were followed during the testing process:

Prior to initiating the uniaxial compression test, the bottom spherical seat was adjusted to ensure a horizontal position of the lower pressure plate. The lubricant was applied to the testing machine’s upper and lower pressure plates. Subsequently, the cube specimen was placed at the center of the pressure plate, as depicted in Figure 3a. The specimen was then leveled to ensure uniform compression. The testing machine was operated to lower the upper-pressure plate until it was approximately 2–3 mm away from the specimen. The oil pump was started, and the testing machine was initiated with a loading rate set at a displacement control of 3 mm/min. The official test commenced and continued until the failure of the specimen, as illustrated in Figure 3b. The failure load was recorded, and the average of three specimen loads was calculated as the compressive strength test result for that particular group of specimens. The stress was determined by dividing the test load by the compressed area of the specimen. At the same time, the strain was obtained by dividing the displacement recorded during the test by the height of the specimen.

The internal structure of vegetation-type concrete differs from that of conventional concrete due to its unique composition with continuous multiple pores, resulting in distinct patterns of failure. The failure mode of conventional concrete is commonly characterized as “fracture,” whereas vegetation-type concrete exhibits a “fragmentation” failure mechanism. In conventional concrete specimens, numerous fracture surfaces are observed post-failure, with the fractured elements predominantly assuming a flake-like structure. In contrast, specimens of vegetation-type concrete display loose granular fragments upon failure.

#### 2.3.2. Effective Porosity

**Figure 4:**
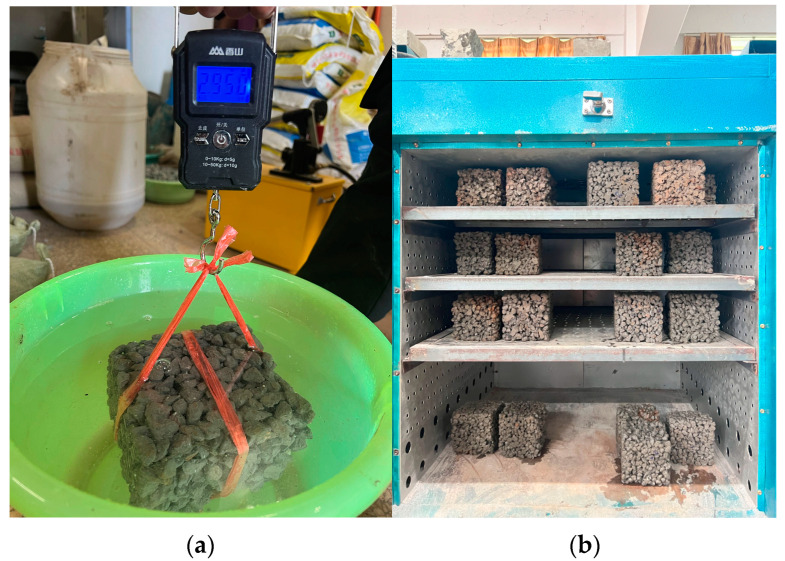
Measuring process of effective porosity of planting concrete: (**a**) immersed water mass m_1;_ (**b**) dry to constant weight mass m_2_.

The formation of pores in vegetated concrete primarily occurs due to voids accumulating between aggregates, and porosity plays a critical role in facilitating plant root growth by providing access to water and nutrients. However, the definition of porosity in the context of porous concrete lacks clarity. In the study conducted by Ghafoori and Dutta [28], it was observed that porosity can be defined in various ways. The measurable voids involved in fluid migration are called porosity, while the total measurable voids encompassing the gaps between aggregates and entrapped air in the cement slurry are termed air content. In this research, measurable voids were defined as effective porosity to ensure clarity in terminology. Initially, the vegetated concrete specimens underwent a 30-day curing period. The specimens’ length, width, and height were measured, and the volume (V) was calculated accordingly. The mass (m_1_) of the specimens was then determined by weighing them after soaking in water for 24 h. Subsequently, the specimens were dried until a constant weight was achieved, and the mass (m_2_) was measured using a portable electronic scale, as depicted in Figure 4. The average value of the three specimens was considered the final test result to minimize measurement errors.

## 3. Test Results and Discussion

### 3.1. Impact of Porosity on Mechanical Properties of Vegetated Concrete

The targeted porosity levels (22%, 24%, 26%, and 28%) in the design of vegetated concrete are influential factors, and the absolute volume method is employed during the mix design process. Uniaxial compression tests were conducted on three specimens in parallel from the experimental group (specimens subjected to vegetation experiments) and the control group (specimens without vegetation experiments). The average values were computed to derive the results of the 30-day uniaxial compression tests, as presented in Table 3. The findings depicted in Table 3 signify that porosity significantly affects the 30-day compressive strength, elastic modulus, and peak strain of vegetated concrete. As the designed porosity increased, there was a gradual decline in compressive strength and elastic modulus. At the same time, the peak strain of vegetated concrete exhibited a substantial increase compared to regular concrete, ranging from 3 to 5 times higher. This observed trend can be attributed to the higher porosity of vegetated concrete, which promotes the occurrence of internal cracks and exacerbates internal damage. Notably, at the 30-day mark, the difference in compressive strength between the vegetated concrete specimens without grass seeds and the composite specimens with grass seeds was insignificant.

#### 3.1.1. Stress–Strain Full Curves

**Figure 5:**
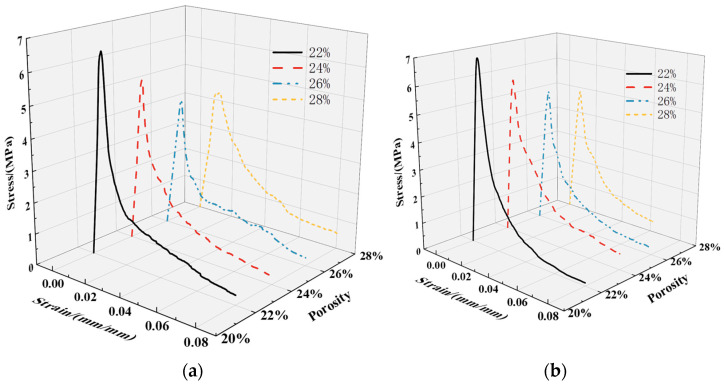
Stress–strain curve of plant-grown concrete under design porosity: (**a**) experimental group; (**b**) control group.

**Figure 6:**
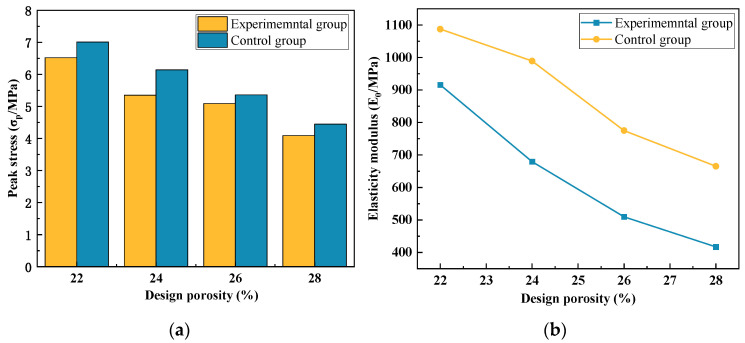
The experimental group was compared with the control group under the design porosity: (**a**) peak stress; (**b**) elasticity modulus.

As depicted in Figure 5, the stress–strain curves of the specimens in each group exhibited distinct characteristics: the ascending segment corresponding to the elastic and elastic–plastic stages and the descending segment corresponding to the failure stage after reaching the peak stress. Figure 6a illustrates the correlation between the designed porosity and peak stress; in contrast, Figure 6b illustrates the correlation between the designed porosity and elastic modulus. Through a comprehensive comparison and analysis of the stress–strain curves among the different groups, the following observations can be summarized:

The uniaxial compression stress–strain curve of vegetation-type concrete generally consists of three stages. Through comparison and analysis of stress–strain curves from various groups, the following characteristics are identified. (1) Initial elastic growth stage: In the initial loading phase, the curve is in the initial stage with a relatively constant slope, indicating a linear stress–strain relationship. Cracks have not yet appeared; (2) Elastic–plastic stage: Following the initial elastic growth, when the load reaches 60% to 80% of the maximum failure load, cracks begin to form around the internal pores of the specimen and continue to expand. At this point, the slope of the curve decreases, cracks rapidly propagate along the weak areas between pores, and the load continues to increase until reaching the peak point; (3) Unstable descent stage: The stress–strain curve bends downward, and the concavity changes, indicating a “turning point.” Due to the interfacial failure between cement and aggregates, cracks expand but the rate is much slower than before reaching the ultimate load. Eventually, a conical failure shape is formed, and the specimen exhibits a dumbbell shape.

Observations of the failure morphology reveal that the failure is characterized by the separation of the cement paste interface bonding each coarse aggregate, with no fracture occurring in the aggregates, which play a role in bearing and transmitting forces. Specimens with larger porosity tend to exhibit fluctuations in the descent stage, especially in groups A_1–3_, A_2–3_, A_1–4_, and A_2–4_. This is attributed to the larger porosity in vegetation-type concrete, leading to a reduced cement volume per unit volume and weakening of the bond strength between aggregates. Uneven deformation is more likely to occur in the descent stage.

#### 3.1.2. Effective Porosity

**Figure 7:**
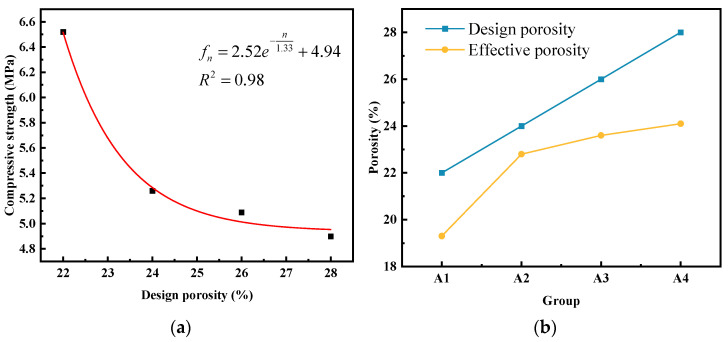
Effect of design porosity on the performance of plant-based concrete: (**a**) the porosity of 30-day compressive strength fitting curve was designed; (**b**) design porosity versus effective porosity diagram.

The influence of designed porosity on the compressive strength and effective porosity of vegetated concrete is illustrated in Figure 7. Within the varying range of porosity from 22% to 28%, the compressive strength of the experimental group decreased from 6.52 MPa to 4.9 Mpa at a 30-day age. A non-linear curve fitting was performed with the designed porosity as the independent variable and the 30-day compressive strength as the dependent variable, and the fitted curve is depicted in Figure 7a. The fitted regression relationship is expressed as follows:

(1) $$f_{n}=2.52e^{-\frac{n}{1.33}}+4.94$$

Increasing the cement content reduces the porosity of vegetation-type concrete when the aggregate particle size remains constant. During the formwork installation, overly dense aggregate stacking can result in the occupation of initially reserved continuous pores by the cementitious material, leading to measured values lower than the design values. The comparison between the design porosity and effective porosity is depicted in Figure 7b. As shown in the figure, the strength of vegetation-type concrete decreases with an increase in porosity. This phenomenon arises from the fact that, in the mix design calculation, the left side of the absolute volume equation comprises three parts: pore volume, cement paste volume, and aggregate volume. Generally, the aggregate volume can be determined based on its fundamental mechanical properties. In this scenario, variations in the reserved pore volume significantly affect the dosage of cementitious material. With an increase in the design porosity, the thickness of the paste surrounding the aggregate and the area of Interfacial bonding between aggregates decrease. Excessive porosity may lead to inadequate strength, while low porosity results in high strength but is unfavorable for plant development. Therefore, maintaining a stable porosity is crucial [29].

### 3.2. Influence of Cementitious Material Ratio on Mechanical Properties of Vegetated Concrete

Using the cementitious material ratio as the influencing factor and the volume method to determine the amount of each material, different cementitious material ratios (4:1, 5:1, 6:1, 7:1) were selected for the experimental and control groups. The results of the 30-day uniaxial compression tests are shown in Table 4. Regarding the stress–strain curve of vegetation-type concrete, different cement-to-aggregate ratios exhibit minimal influence on the ascending portion and peak strain but they do exert a certain impact on the descending portion. With an increase in cement content, the slope of the descending portion also increases. In conjunction with the analysis of failure patterns, vegetation experiments contribute to a certain extent to mitigating the brittle failure of concrete.

#### 3.2.1. Stress–Strain Curves

**Figure 8:**
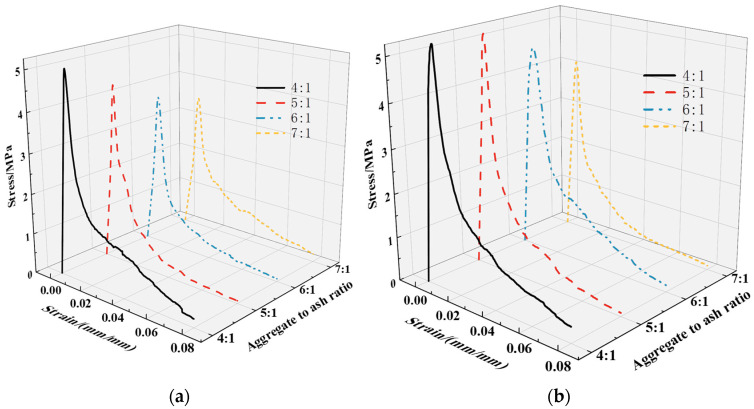
Stress–strain curve of plant-formed concrete under different aggregate-to-cement ratios: (**a**) experimental group; (**b**) control group.

**Figure 9:**
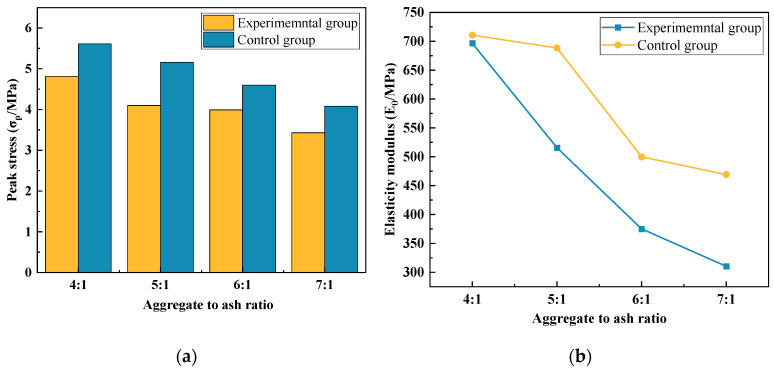
Ash collection ratio between experimental group and control group is compared: (**a**) peak stress; (**b**) elasticity modulus.

As depicted in Figure 8, the stress–strain curves under different cementitious material ratios exhibit similar characteristics to those observed for the design porosity. Throughout the entire loading process, a continuous sequence of “microcracks appearing internally → gradual crack propagation → connectivity → extension → loss of bearing capacity → specimen failure” is observed. Figure 9a illustrates the correlation between the design cementitious material ratio and peak stress. In contrast, Figure 9b depicts the correlation between the cementitious material ratio and elastic modulus.

#### 3.2.2. Effective Porosity

**Figure 10:**
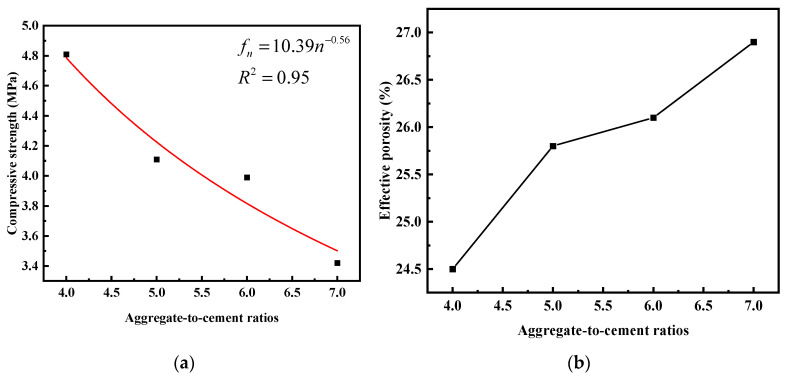
Influence of ash ratio on performance of plant-grown concrete: (**a**) aggregate-to-cement ratios—30-day compressive strength fitting curve; (**b**) effective porosity.

Based on the pre-experimental results for the design porosity, the target porosity was determined to be 26%. The impact of the cementitious material ratio on the compressive strength and effective porosity of vegetated concrete is illustrated in Figure 7. A non-linear curve fitting was performed with the cementitious material ratio as the independent variable and 30-day compressive strength as the dependent variable. The fitted curve is presented in Figure 10a, and the regression relationship satisfies:

(2) $$f_{n}=10.39n^{-0.56}$$

Results indicate that under the same water-to-cement ratio, with an increase in the cementitious material ratio, the porosity of vegetated concrete increased, and the 30-day compressive strength decreased. The effective porosity is illustrated in Figure 10b. When the cementitious material ratio was relatively low, an increase in cement content led to a thicker mortar layer encapsulating the aggregates and a larger bonding area between aggregates. Consequently, the structure of vegetated concrete exhibited its ultimate strength, failing.

### 3.3. Correlation between Compressive Strength and Effective Porosity

**Figure 11:**
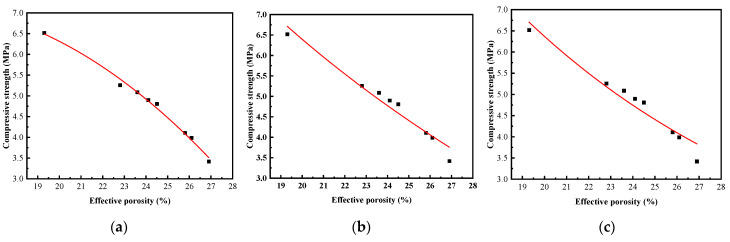
Fitting of compressive strength and effective porosity curve: (**a**) polynomial function; (**b**) logarithmic function; (**c**) exponential function.

Numerous experiments have extensively investigated the correlation between porosity and strength in diverse porous materials [30]. Hasselmann et al. [31] proposed a polynomial equation that can be applied to describe the correlation between porosity and strength in materials. Schiller et al. [32] suggested utilizing a logarithmic relationship to depict the connection between porosity and strength in materials. Ryshkevitch et al. [33] advocated for an exponential relationship as a preferred method for describing the correlation between porosity and strength in porous materials. In this study, a regression analysis was conducted on the 30-day test results of vegetated concrete. Three types of functions were selected for fitting, aiming to establish the correlation between effective porosity and 30-day compressive strength. The regression analysis results are presented in Table 5, and the curve fitting is depicted in Figure 11. The specific regression analysis approach is as follows: (1) Establish a scatter plot relationship between effective porosity and compressive strength; (2) refer to existing empirical formulas and select the relationship type based on the scatter plot; (3) utilize the least squares method to determine the parameters; (4) use correlation tests to ascertain the effectiveness of each formula. Results indicate that among the three relationship equations, the polynomial equation $y=A+B_{1}x+B_{2}x^{2}$ has an R^2^ of 0.99, making it the most suitable equation for describing the correlation between effective porosity and compressive strength.

### 3.4. Vegetative Experiment of Vegetated Concrete

The variation in effective porosity plays a crucial role in determining the available space for plant root growth within vegetated concrete. The bonding of aggregate particles by a thin layer of hardened cement slurry is a crucial factor, and an increase in cement content can result in excessive alkalinity, which is unfavorable for plant growth in later stages. The findings revealed a decrease in concrete compressive strength by 3–10 MPa after 30 days, and the increase in porosity counteracted the weakening effect of plants on strength. Therefore, it is vital to conduct vegetation experiments to identify suitable plant species for growth in areas with red soil.

**Figure 12:**
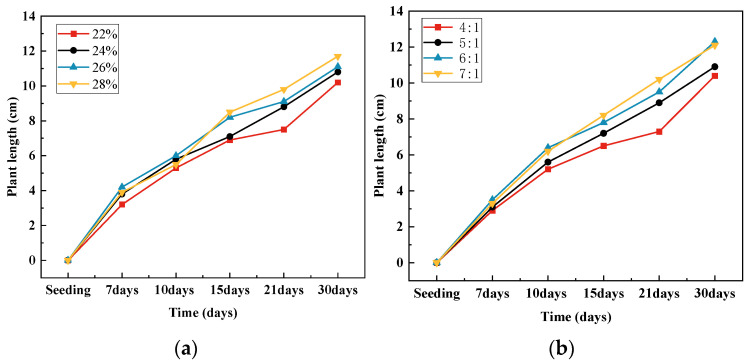
Thirty-day planting test curve: (**a**) design porosity; (**b**) ash collection ratio.

The vegetation planting in this experiment was conducted in April, considering the favorable germination conditions provided by the average temperature of 19 °C in the Kunming region during that month. White Clover exhibited germination on the fourth day, with plant heights ranging between 1 and 1.5 cm after thirty days. Ryegrass, Bermuda Grass, and Fountain Grass sprouted and grew after 6 days of sowing, reaching plant heights of 5 to 6 cm after 10 days, with root lengths of approximately 1 cm. Ryegrass demonstrated strong resistance to alkaline conditions and thrived on the vegetated concrete. After 30 days of sowing, ryegrass reached a plant height ranging from 10 to 12 cm. The growth curves of the 30-day vegetation experiment under these parameters are depicted in Figure 12. The upper parts of the plants exhibited rapid growth, and the root systems developed well. By the end of the 30 days, the plants naturally grew to 10 cm, with roots extending 4 cm, enabling penetration through a 3-centimeter-thick vegetated concrete specimen.

**Figure 13:**
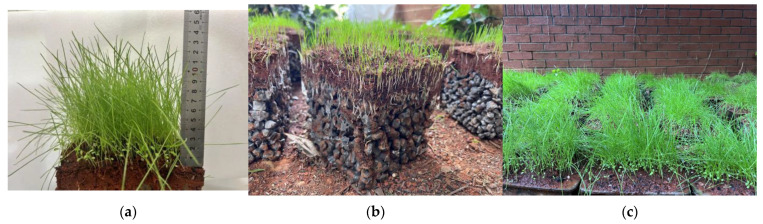
Growth of 30 days of planting test: (**a**) growth height; (**b**) plant 10-day root growth; (**c**) Vegetation appearance.

For vegetation concrete made with ordinary Portland cement, the pH value tends to be relatively high, which may not provide optimal growth conditions for ryegrass. Therefore, through mixed design, it was essential to establish an appropriate porosity level that creates an internal environment conducive to plant growth while meeting the strength requirements of the vegetation concrete. Figure 13 visually presents the growth of plants in this context.

## 4. Conclusions

This study presents a comprehensive analysis of vegetation concrete’s mechanical properties and vegetation growth effects, yielding significant findings. The specific conclusions derived from the study are as follows:

1. Utilizing aggregate gradation within the range of 10–15 mm and a water–cement ratio ranging from 0.40 to 0.43, a design porosity between 22% and 28% yielded a 30-day compressive strength ranging from 4.9 to 6.52 MPa, effective porosity ranging from 19.3% to 24.1%, and a density ranging from 1748.14 to 2062.22 kg/m^3^. Notably, specimens with higher effective porosity exhibited uneven deformation in the descending segment of the stress–strain curve;
2. Under the same aggregate gradation and water–cement ratio, incorporating a fly ash ratio ranging from 4% to 7% resulted in a 30-day compressive strength ranging from 3.42 to 4.81 MPa, effective porosity ranging from 24.5% to 26.9%, and a density ranging from 1694.81 to 1866.67 kg/m^3^;
3. The investigation of stress–strain curve characteristics for vegetation concrete revealed positive correlations between compressive strength and the elastic modulus with cement content. In contrast, negative correlations were observed with porosity. Peak strain exhibited a positive correlation with porosity and a negative correlation with cement content. Notably, the peak strain for vegetation concrete was 3–5 times greater than ordinary concrete. A fitting analysis was conducted on the relationship between the compressive strength and effective porosity of vegetation-type concrete after 30 days, revealing that a polynomial function adequately captures the correlation between compressive strength and effective porosity;
4. Based on the vegetation effects observed at the 30-day mark, ryegrass demonstrated faster growth than other grass species and exhibited some adaptability to alkaline soil conditions. Within 10 days, the plant height reached 3–4 cm, while the root length measured approximately 1 cm. After 30 days, the root length extended to 4 cm, capable of penetrating a 3-centimeter-thick vegetation concrete specimen.

## Figures and Tables

**Table 1:** The primary chemical constituents of cement.

Test Indicators	SO_3_/%	Cl^−^/%	Ignition Loss/%	Retained on the 80 μm Sieve/%
Test results	3.44	0.04	4.37	3.5
Industry standards	>3.5	>0.06	—	<10.0%

**Table 2:** Mix ratio table of plant-growing concrete.

Sample Number	Factors		Mix Proportions		
	Design Porosity	Aggregate Cement Ratio	Aggregate kg/m^3^	Cementitious Material kg/m^3^	Water kg/m^3^
Group1 (Design porosity)					
1–1	22%	4.2:1	1460	345	124
1–2	24%	4.6:1	1460	316	114
1–3	26%	5.1:1	1460	287	103
1–4	28%	5.7:1	1460	258	93
Group (Aggregate cement ratio)					
2–1	26%	4:1	1336	334	134
2–2	26%	5:1	1430	286	114
2–3	26%	6:1	1500	250	100
2–4	26%	7:1	1561	223	89

**Table 3:** The data from the 30-day uniaxial compression tests under different designed porosity levels.

Group	Number	Design Porosity	Compressive Strength/MPa	Modulus of Elasticity/MPa	Peak Strain
Experimental group	A_1–1_	22%	6.52	915.67	0.00742
	A_1–2_	24%	5.26	679.32	0.00759
	A_1–3_	26%	5.09	509.64	0.00876
	A_1–4_	28%	4.90	417.06	0.01164
Control group	A_2–1_	22%	7.01	1087.54	0.00661
	A_2–2_	24%	6.14	989.25	0.00765
	A_2–3_	26%	5.36	775.15	0.00781
	A_2–4_	28%	4.45	665.26	0.00989

**Table 4:** Thirty-day uniaxial compression test data under different aggregate-to-cement ratios.

Group	Group	Aggregate-to-Cement Ratio	Compressive Strength/MPa	Modulus of Elasticity/MPa	Peak Strain
Experimental group	B_1–1_	4	4.81	696.55	0.00819
	B_1–2_	5	4.11	515.56	0.00831
	B_1–3_	6	3.99	375.08	0.00852
	B_1–4_	7	3.43	310.34	0.00928
Control group	B_2–1_	4	5.61	710.52	0.00622
	B_2–2_	5	5.16	688.50	0.00716
	B_2–3_	6	4.60	499.84	0.00928
	B_2–4_	7	4.08	469.08	0.00944

**Table 5:** Regression analysis of compressive strength and effective porosity.

Category	Equation	The Fitted Equation	R^2^
Polynomial function	$y=A+B_{1}x+B_{2}x^{2}$	$y=3.64+0.53x-0.02x^{2}$	0.99
Logarithmic function	y=Aln(x)+B	y=−8.9ln(x)+33.06	0.95
Exponential function	$y=Ae^{Bx}$	$y=27.73e^{-0.07x}$	0.93

**Note:** *x*: Effective porosity; *y*: the 30-day compressive strength; *A*, *B*_1_, *B*_2_: Constants.

## Data Availability

Data are contained within the article.
